# A taxonomy of risk‐associated alternative health practices: A Delphi study

**DOI:** 10.1111/hsc.13386

**Published:** 2021-05-26

**Authors:** Bernie Garrett, Timothy Caulfield, Blake Murdoch, Matt Brignall, Atul Kumar Kapur, Susan Murphy, Erin Nelson, Jillian Reardon, Mark Harrison, Jonathan Hislop, Barbara J. Wilson‐Keates, Joseph Anthony, Peter S. Loewen, Richard M. Musoke, Joan Braun

**Affiliations:** ^1^ School of Nursing University of British Columbia Vancouver BC Canada; ^2^ Faculty of Law Health Law Institute University of Alberta Edmonton AB Canada; ^3^ Puget Sound Family Health Tacoma WA USA; ^4^ Emergency Medicine University of Ottawa Ottawa ON Canada; ^5^ Department of Physical Therapy Faculty of Medicine University of British Columbia Vancouver BC Canada; ^6^ Faculty of Pharmaceutical Sciences University of British Columbia Vancouver BC Canada; ^7^ Centre for Health Evaluation and Outcome Sciences (CHEOS) St. Paul’s Hospital Vancouver BC Canada; ^8^ Family Medicine University of British Columbia Vancouver BC Canada; ^9^ Faculty of Health Disciplines Athabasca University Edmonton AB Canada; ^10^ School of Population and Public Health University of British Columbia Vancouver BC Canada; ^11^ Bora Laskin Faculty of Law Lakehead University Thunder Bay ON Canada

**Keywords:** alternative health care, complementary and alternative health, complementary and alternative medicine, Delphi study, risk

## Abstract

Defining alternative health care and the recording of associated adverse events and harm remains problematic. This Canadian study aimed to establish and classify risk‐associated alternative health practices in a Delphi study undertaken with an interdisciplinary panel of 17 health experts in 2020. It provides a new functional definition of alternative health care and an initial taxonomy of risk‐associated alternative health care practices. A number of risk‐associated practices were identified and categorized into general practices that conflict with biomedical care or largely untested therapies, alternative beliefs systems, physical manipulative alternative therapies, and herbal and nutritional supplements. Some risk significant harms including major physical injuries or even death. The lack of systematic methods for recording adverse events in alternative health care makes establishing the frequency of such events challenging. However, it is important that people engaging with alternative health care understand they are not necessarily risk‐free endeavours, and what those risks are.

**What is known about this topic?**

There is a public perception that the use of alternative health care is safe and has low risk, and evidence on the nature of risk in alternative health is lacking. Evidence supports the view that particular personality traits make engagement with alternative health care more likely.

**What this paper adds?**

A new functional definition of alternative health care, an initial taxonomy of risks associated with alternative health care practices, and a consensus amongst a range of diverse health professionals on the types and severity of risks encountered in alternative health care.

## 1 INTRODUCTION

The alternative health care sector has grown significantly in the economically developed world over the past two decades, prompting research into the possible motivators and factors associated with its uptake (Barnes et al., 2008; Sirois et al., 2016). Far from being a cottage industry, it now represents a major economic sector. The use of alternative medicine or health care may be positive or benign, but there is increasing evidence that people are engaging in more hazardous alternative health behaviours, such as ignoring effective medical treatments in preference to alternative health care, or utilizing unsafe or experimental therapeutics (Flood, 2017; Simon, 2019; Ventola, 2010). A recent Yale study found that cancer patients using alternative health care in favour of medicine contributed to higher death rates (Johnson et al., 2018a). More extreme examples of high‐risk alternative health care make the news headlines, such as the case of the Alberta couple whose son died of meningitis after being treated with natural remedies (Aldach, 2016).

Levels of risk in any form of health care are difficult to quantify, but a significant clinical risk may be considered one where unnecessary harm is a likely outcome of a health care practice (taking into consideration current knowledge, available resources and the context of care delivery). This must also be compared to the risk of non‐treatment or another treatment (Canadian Institute for Health Information, 2016). To date, there has been little research exploring the nature of risk associated with the uptake of alternative health care.

### 1.1 Background

Alternative health care has proven difficult to define and has been described as therapeutic interventions arising from alternative traditions, or based upon a metaphysical spiritual basis, or asserting empirically unverified theories involving the manipulation or effects of theoretical energies or matter (Ernst & Cassileth, 1998; Offit, 2012; Thorne et al., 2002).

A 2018 U.K. Ipsos MORI survey found visits to complementary and alternative medicine (CAM) practitioners (such as acupuncturists, chiropractors and naturopaths) had risen from 12% of the population in 2005 to 16% by 2015 (Sharp et al., 2018). Another 2016 report indicated that Americans spent more than $30 billion on alternative therapies. This included a wide range of therapies and remedies such as homeopathy, chiropractic, Reiki, acupuncture and nutritional supplements. This publicly funded U.S. report, released jointly by the government National Centre for Complementary and Integrative Health (NCCIH) and the Centres for Disease Control and Prevention, found that around 17% of Americans had sought out some type of alternative therapy in the last year (Nahin et al., 2016). Studies have also shown that 70%–80% of Canadians have used CAM at least once if not regularly, spending over $8 billion on them (Esmail, 2017; Health Canada, 2003). This included $6.5 billion spent on provider services such as homeopathy, chiropractic, Reiki, acupuncture and another $2.3 billion spent on herbs, vitamins, special diet programmes, equipment and literature in 2016 alone (Esmail, 2017). It has been suggested that the global alternative health care market will be worth $210 billion by 2026 (Grand View Research, 2019).

This rapid growth and commercialization of alternative health care has led to much research into the possible factors associated with the use of CAM. Research suggests, rather than being used by people living alternative lifestyles, alternative health care users are mainly female, are well‐educated, employed and often those with chronic health issues (Barnes et al., 2008; Bromfield & McGwin, 2013; Foltz et al., 2005; Sirois et al., 2016). People with chronic illnesses that conventional medicine cannot cure are particularly challenged with the ongoing process of self‐care management and so often seek alternative options. The supplementary use of alternative medicine (complimentary) is well‐known here. For example, people with inflammatory bowel disease (IBD), multiple sclerosis (MS), diabetes, arthritis and cancer are all significant users of alternative medicine, with reported usage rates of 52% for IBD, 57% for MS and diabetes, 66% for arthritis and as high as 80% for cancer (Bernstein & Grasso, 2001; Garrett, et al., 2019; Kaboli et al., 2001; Nayak et al., 2003; Rao et al., 1999; Sirois, 2008; Yeh et al., 2002).

Although alternative health care is often regarded as harmless, serious injuries and deaths do occur with its use, just as with conventional medicine (Johnson et al., 2018a; Nielsen et al., 2017; Offit, 2013; Paulus & Belill, 2018). The most obvious form of harm is that of direct damage resulting from the use of an alternative therapy, but there are also other forms of harm. Harm may be considered as anything that has a negative effect on the welfare of participants, and the nature of the harm may be social, behavioural, psychological, physical or economic. Unlike publicly funded health care systems, the recording of adverse events and harm in alternative health care remains disjointed and ill‐described, as it is mainly practiced in private settings and often reported ad hoc or under less regulated conditions. Hence, the absence of good quality research on the incidence and severity of any unfavourable effects of alternative therapies currently makes it impossible to quantify risk probability in precise terms.

Nevertheless, there is some work exploring the psychology of illness and decision‐making behaviours that substantiates theories that there are specific personality traits that may help explain engagement with risk‐associated alternative health care practices, such as beliefs about self‐control and negative beliefs about science (Barnes et al., 2008; Bishop et al., 2007; Furnham, 2013; Garrett et al., 2019; Sirois et al., 2016). Generally, work remains relatively undeveloped, and this study seeks to build knowledge and theory in this emerging field, to establish an initial taxonomy of the kinds of risk‐associated health care people are engaging with.

This area is understudied, and the actual nature of risks involved in this area are not well understood and difficult to quantify. The aim here was not to undertake a comparative study with biomedicine, nor an examination of socio‐political motivations, or the influence of biomedical power structures, geographic origins of therapeutic approaches, their ontological premises, geo‐political or social power relationships in health care. The aim was simply to establish whether they posed any risks to people using them, and if so, what those were in practical terms based on existing evidence.

The study aimed to establish the nature of this growing phenomenon in order to explore the social psychology supporting the uptake of alternative health care risk‐associated practices in future work. As an exploratory study to address the current gap in understanding, this work was focused upon addressing the following questions:

1. Do established definitions of CAM/health work as a practical way to identify them and potential associated risks?
2. What types of risk‐associated alternative health practices are apparent?
3. Can potentially risk‐associated alternative health behaviours be identified and classified in a systematic taxonomy?

## 2 METHOD

The grounding assumptions for this work included acceptance of the value of a scientific and evidence‐based practice (EBP) approach to health care arising from the panel's desire to provide a broad context for this work in empirical client‐centred practice (Sackett et al., 2000). A further assumption was that science‐based health care represents the current basis for the majority of public health care practice in most economically developed countries. Whilst much of the world utilizes alternative frameworks, and there are ontological arguments on the nature of being, health and humanity, these form the basis of alternative health belief systems outside of a scientific paradigm.

**FIGURE 1:**
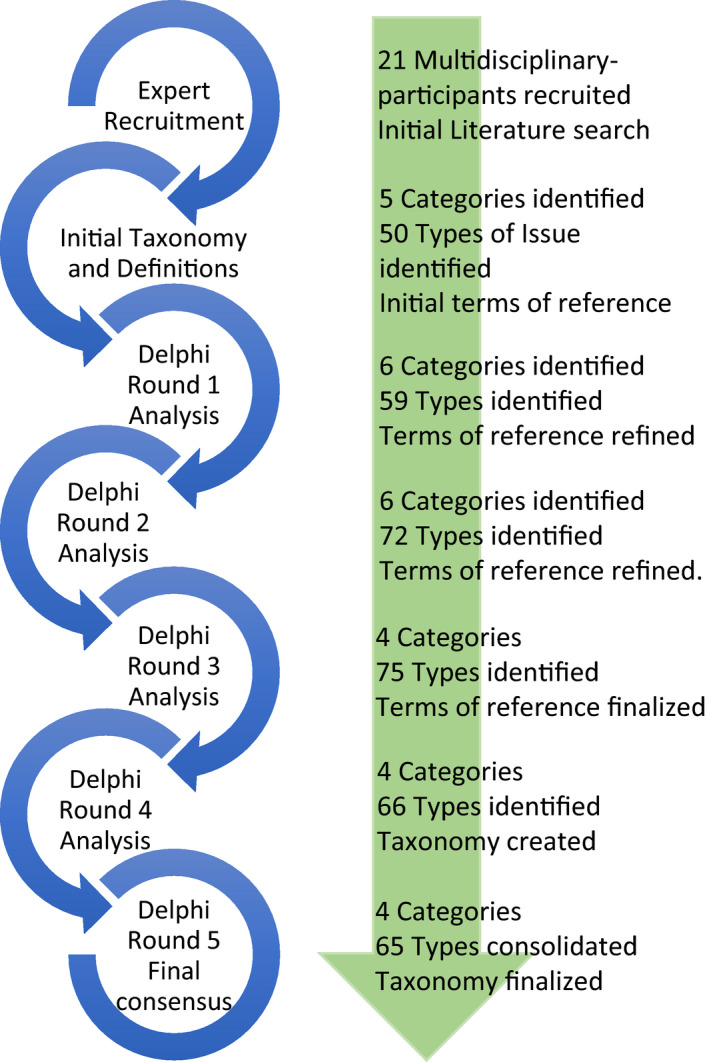
The Delphi process [Colour figure can be viewed at wileyonlinelibrary.com]

Ethical approval was obtained from University of British Columbia Behavioural Research Ethics Board before recruitment commenced, and the work carried out between September 2019 and August 2020. All participants provided written consent. A Delphi approach was selected to develop an expert consensus on alternative health care risks (Powell, 2003). The Delphi method allows equal participation of experts from different disciplines to provide input about their experiences (Habibi et al., 2014; Powell, 2003; Keeney et al., 2011). An expert panel of health professionals was recruited for consultation and structured feedback to arrive at a consensus. The process started with participant recruitment followed by five rounds of expert consultation and feedback (Figure 1). The Delphi panel was used to develop a consensus on risk‐associated alternative health care practices they had observed or were aware of, identifying the specific harms associated with each, and categorize them in a taxonomy assessing the level of risk for each.

### 2.1 Phase 1: Expert recruitment

Recruitment was purposeful through an advertisement for participation to experienced health professional clinicians and researchers in universities and by chain sampling, where participants referred the invitation to other suitably qualified candidates. The inclusion criteria were for clinical, legal, social health care professionals and researchers with at least 10 years of experience and an identified interest in alternative health care, with the aim to create a diverse range of professionals, including alternative health care practitioners.

It is recommended that Delphi studies should have a minimum of five participants to obtain a sufficient range of views (Clayton, 1997; Habibi et al., 2014; Sinclair et al., 2016). The objective is not to have a sample that is representative of the population, but a sample of those with expertise and interest in a particular phenomenon (Cohen et al., 2011; Hasson et al., 2000; Powell, 2003). Therefore, purposeful and self‐selection is a necessary element (Keeney et al., 2011; Powell, 2003). A multidisciplinary team of 21 health and legal professionals was established, with the final team consisting of four physicians, four nurses, three pharmacists, three physiotherapists, two social workers, two lawyers (with expertise in harm, injury and case law), an epidemiologist, a naturopath and a chiropractor. This balance of professions was purposefully designed to achieve a broad range of views and expertise of different health and legal professionals. Primarily the focus was upon those working within the Canadian public health sector, and biomedical and psycho‐social practitioners. During the course of the study, four participants withdrew due to work‐related commitments or for health reasons, including one physician, one nurse, one physiotherapist and one social worker (*n* = 17).

### 2.2 Phase 2: Identification of potential alternative health care harm

The U.S. NCCIH framework was selected as an initial basis for categorization to classify adverse alternative health issues from the literature. This framework is widely accepted and is currently used by the Cochrane Collaboration to guide reviews and systematically defines and classifies alternative health and complementary practices (Susan Wieland et al., 2011).

An initial literature review was undertaken involving the search of major electronic databases for published journal articles, legal cases and media reports that involved harm with the use of alternative health care. Online databases searched included, PubMed, MEDLINE, CINAHL Embase, the CanLII legal database and Google/Google Scholar. Search terms included the terms and stems derived from Table 1.

**TABLE 1:** Search terms derived from the NCCIH categorization

Alternative health/medicine belief systems	Traditional Chinese medicine (including acupuncture), homeopathy, naturopathy, Ayurvedic medicine, indigenous and traditional medicine systems
Physical manipulative interventions	Massage, chiropractic, reflexology, hydrotherapy and craniosacral therapy
Herbal and nutritional interventions	Herbal remedies, vitamins, dietary supplements, diets, aromatherapy and detoxification therapies
Mind‐body interventions	Meditation, guided imagery, hypnotherapy, music therapy, bio or neurofeedback, yoga, Tai Chi, Qigong and dance therapy

Abbreviation: NCCIH, National Centre for Complementary and Integrative Health.

These search terms were further meshed with the following: ‘harms, risks, death, damage, injury’ and ‘adverse effects/events’ resulting in 1,765 initial citations. Additional practices were subsequently identified through an initial request to the panellists who reported observed cases in their practice related to use of alternative health care. Media reports of harm associated with alternative health care were only included if there was additional supporting evidence referenced. The results of this activity informed construction of an initial listing of the main types of risk‐associated alternate health activities which was sent out to the Delphi expert panel.

### 2.3 Phases 3–5: Delphi questionnaire rounds—Taxonomy development

Risk‐associated alternative health care practices as initially identified from the literature review of published academic case reports, legal and media reported cases were refined further through iterative consultation rounds. Five rounds of consultation, feedback and analysis occurred. In the first round, a draft terms of reference document and simple categorization and listing of alternative health care risks based on the NCCIH framework in a spreadsheet was emailed to the panel. They were instructed to review the project terms of reference, definitions, listed items, categorization and the rationale provided for inclusion. They were asked for feedback on whether they agreed with the items, had additional items to include, to review the categorization used and make suggestions for improvement. Additionally, they were asked to indicate the level of risk that they thought was associated with each item and add any relevant citations. A turnaround period of 3 weeks for each phase was given to review the emailed documents and provide feedback.

The feedback and suggestions provided by each panellist were collated by two research assistants. Revisions were made to the draft taxonomy, working definitions, rationales, harms, levels of risk and the revisions sent back to participants. Panellists reviewed the changes and provided additional feedback in order to attain a consensus about the guiding definitions and taxonomy.

## 3 RESULTS

### 3.1 Creation of the working definitions and taxonomy

Using the NCCIH framework proved problematic for creating a logically consistent system to categorize practices because of imprecise criteria, conflation and non‐exclusive categorizations. Therefore, it was simplified into a functional framework as described in Table 2.

**TABLE 2:** Harm types

Direct harm	Harm resulting from prescribed substances (including self‐prescribed), Harm resulting from procedures, Harm resulting from reducing the effectiveness of, or causing detrimental effects from existing medical therapies, Economic harm with financial loss through payment for ineffective interventions.
Indirect harm	Harm resulting from replacing established effective care, Harm resulting from delay of treatment or failure to diagnose a medical problem and disease progression, Harm resulting from accepting detrimental health advice beyond the scope of the practitioner's abilities, educational preparation/training and clinical experience, Economic harm: negative effects on personal finances (impacting budget available for other needs), and social impact of lost work productivity.

Arriving at a consensus on harms and risks also proved challenging, as the group had diverse opinions. Harm was accepted as involving injury to, or impairment of, physical or mental health or financial condition, including injury suffered as a result of knowing of another person suffering ill treatment. The consensus was that risk involved both the probability of exposure to danger and its significance, whilst harm is the outcome associated with exposure. Identified harms were categorized as either direct or indirect (Table 2).

An absence of good‐quality data and research on the incidence of any adverse effects of alternative therapies made it impossible to quantify risk precisely in terms of probability of harm or in meaningful comparative terms as no systematic reporting frameworks existed and much of the activity occurred in private practice or was undocumented. Therefore, quantitative approaches to risk and its public‐perception, such as the work of David Spiegelhalter (Spiegelhalter, 2003), cannot be easily applied here. Therefore, as practical indicators of level of risk, three categories were adopted (higher, moderate and lower) based upon (a) the significance of harm resulting from the practice, (b) the existence of verified case examples (rather than incidence) and (c) potential consequences versus demonstrated value of the therapy in question (Table 3).

**TABLE 3:** Alternative health care risks: Levels of risk

*Higher*: A higher‐risk alternative practice or therapy is one that exposes the person seeking the therapy (or others) to a risk of serious and/or permanent physical, psychological harm or death. Cases of harm and deaths have been reported in the clinical literature, with practice and legal cases as a result of use of the therapeutic identified. Occurrences may be rare, but the potential consequence of serious harm or death are considered sufficient to outweigh any asserted potential value of the therapy.
*Moderate*: A moderate‐risk alternative practice or therapy is one that exposes the person seeking the therapy or others to a risk of (potentially reversible) significant physical, psychological or economic harm. This includes the potential for causing significant financial loss to an individual or community. Cases of harm have been reported in the clinical literature, with practice and legal cases as a result of use of the therapeutic identified. Occurrences may be rare, but the potential consequences of harm may outweigh the asserted potential value of the therapy.
*Lower*: A lower‐risk alternative practice or therapy is one that exposes the person seeking the therapy or others to some short‐term risk of physical, psychological or economic harm. This includes the potential for causing financial loss to an individual or community. Cases of harm have been reported in the clinical literature, with practice and legal cases as a result of use of the therapeutic identified. Occurrences may be rare, but indicate in some instances using it may be harmful.

In the initial three rounds, six categories with 75 types of risk‐associated activities were identified. Further rounds led to the merging some of categories and the removal of some items due to insufficient evidence. Finally, four core categories and 65 types of activity were identified (Table 4) supported by at least three citations of evidence, as detailed in Table 5. For each item, we identified the category, issue, rationale and type of associated harm.

**TABLE 4:** Alternative health care risk major categories identified

1. General risks	General risk‐associated alternative health practices that have been identified across a broad range of alternative health care activities
2. Risks with complete alternative health belief systems	Any specific risk‐associated practices identified in complex complete systems of non‐biomedical science‐based health beliefs (including spiritual/human energy belief systems), for example, traditional Chinese medicine, homeopathy, naturopathy, Ayurvedic medicine, Reiki, and therapeutic touch
3. Risks with alternative physical manipulative therapies and interventions	Any specific risk‐associated practices identified in therapies involving physical manipulative, or mind‐body interventions, for example, chiropractic, reflexology, craniosacral therapy, yoga and Tai Chi
4. Risks with alternative herbal and nutritional interventions	Any specific risk‐associated practices outside of complete alternative belief‐systems that involve the use of herbal remedies, nutritional supplements, diets, and/or detoxification therapies and aromatherapy

**TABLE 5:** Taxonomy of alternative health care risk‐associated behaviours

Issue	Rationale	Type of harm	Level
1. General activities			
Using alternative health care instead of the existing biomedical standard of care for medically treatable conditions	Avoidance of established therapeutic interventions using standards of care that have been demonstrated as effective interventions through scientific study Increased risk of disease progression and lost opportunity if treatment not effective May involve belief in alternative medicine, distrust of medicine or science, costs or availability of medical treatment	Indirect: Harm resulting from replacing established effective care Harm resulting from delay of treatment or failure to diagnose a medical problem and disease progression	Higher
Using alternative therapeutics which are new and where side‐effects are unknown	Most alternative therapeutics are not as well‐regulated as drugs and medical treatments Risk with using untested products and therapies Patients can obtain products online allowing them to bypass Health Canada safeguards	Direct: Harm resulting from prescribed substances (including self‐prescribing) Harm resulting from procedures	Higher
Using alternative therapeutics alongside existing medical treatments without informing the medical provider	Risk due to possible adverse interactions/effects between therapeutics	Direct: Harm resulting from prescribed substances (including self‐prescribing)	Higher
Using alternative health treatments for diagnoses not currently recognized as biomedical illnesses or misdiagnosed (do not meet biomedical diagnostic criteria), for example, candida overgrowth, adrenal fatigue, chronic Lyme disease, etc.	People seek out alternative health care diagnoses for misdiagnosed or more generic chronic health issues, when biomedicine does not meet their needs Exposure to unnecessary alternative health treatments which may have adverse health and financial effects Economic harm with financial loss through payment for ineffective interventions	Direct and indirect: Harm resulting from prescribed substances (including self‐prescribing) Harm resulting from procedures	Moderate
Utilizing alternative health care for the treatment of medical conditions based on misinformation in deceptive advertising/marketing	False claims are more frequently made in alternative health advertising, specifically in that they can treat medical conditions beyond the scope of their practice. This may result in direct or indirect harm to the person, for example, Chiropractic therapy to prevent COVID‐19 infection	Direct and Indirect: Economic harm: Economic harm with financial loss through payment for ineffective interventions Harm resulting from accepting detrimental health advice beyond the scope of the practitioner's abilities/education/experience Harm resulting from reducing the effect of existing medical therapies	Moderate
Taking part in an alternative health research that has not been approved by an independent research ethics board	Exposure to experimental alternative health treatments which may have adverse health effects without informed consent Supervision of alternative health research in private settings is often not subject to the same level of ethical scrutiny as public health research Poorly designed and regulated research using confirmation design studies may be used to legitimize them	Direct: Harm resulting from prescribed substances (including self‐prescribing) Harm resulting from procedures Harm resulting from replacement of established effective care	Lower
Impact of the financial costs associated with the use of alternative practitioners/therapeutics not covered under public health care provision	Financial loss where ineffective alternative health products and services are sold on the basis of false curative claims Highly priced products marketed with misinformation, for example, propriety supplements sold at 10× the cost of generic forms Deceptive advertising (often from offshore sources) causing patients to spend their budget on expensive alternative health products of known inefficacy	Direct or indirect: Economic harm: financial loss through payment for interventions known not to work	Lower
Societal economic impact associated with the use of alternative health care provision when covered by third parties	Financial loss to society due to increased insurance costs where ineffective alternative health products and services are paid for by third‐party health insurance on the basis of consumer demand	Indirect harm: Economic harm: financial loss through payment for interventions known to be ineffective	Lower
2. Alternative health belief systems activities			
(a) Traditional Chinese medicine (TCM)			
Toxicity with specific TCM medicines: Monkshood (Aconitum napellus)* Thunder God Vine (Tripterygium wilfordii)†‡ Jia Yi Jian capsules/tea** Aristolochic Acid (found in: Asarum, Bragantia (Apama or Thottea)^††^ Niuhuang Jiedu Pian†‡ Bak Foong Pills‡‡ Fabao^§^	Can cause: Cardiac arrhythmias, gastrointestinal symptoms, nausea, vomiting, abdominal pain, diarrhoea, respiratory paralysis* Dermatological reactions, haematological reactions (delayed clotting), cardiovascular events, dysmenorrhea†‡ Cardiovascular problems, hypertension** Nephro/hepatotoxicity (e.g., acute renal failure)^††^ Contains realgar (arsenic)†‡ Contains high levels of lead – neurotoxicity, developmental delays, abdominal pain‡‡ Higher levels of mercury – teratogenic, neurotoxicity, muscles spasm, loss motor skills, of sensation^§^	Direct: Harm resulting from prescribed substances (including self‐prescribing)	Higher
Injuries with cupping: Used for a wide variety of health problems, for example, anaemia, arthritis etc.	Bruising and burns (sometimes severe)	Direct: Harm resulting from procedures	Moderate
Injures with TCM acupuncture: Used for a wide variety of health problems, for example, immunological etc.	Adverse effects include infection, trauma, pneumothorax^¤^, or nerve damage^¤^	Direct: Harm resulting from procedures	Higher^¤^ Moderate
Injuries in TCM acupuncture with moxibustion: Treatment of pain, organ related or immunological conditions with acupuncture needling and heat	Burns resulting from treatment	Direct: Harm resulting from procedures	Moderate
TCM acupuncture in vulnerable populations: Older, immunocompromised people, or with chronic respiratory disease	Increased risk of trauma, nerve damage or infection with these populations		Lower
(b) Naturopathic and homeopathic medicine			
Adverse events with naturopathic intravenous (IV) therapies: Use of IV therapies by naturopaths for vitamin supplements, chelation, for a range of conditions including infections, hangover cures and autism	Lack of practitioners' experience with acute and emergency care to deal with adverse reactions Infections resulting from circulatory access, and inadequate administration safety procedures Toxicity risks with rapid direct access to the circulatory system for substance administration Contamination or other preparation issues Use of illegally imported or compounded items circumventing regulatory control	Direct: Harm resulting from procedures Harm resulting from prescribed substances (including self‐prescribing)	Higher
Injuries with naturopathic colonic irrigation therapies: Colon cleansing using the administration of large volumes of water, coffee or other substances by colonic enema	May cause infections, tears or intestinal perforations, cramping, bloating, nausea and vomiting, diarrhoea, dizziness, dehydration and pancreatitis	Direct: Harm resulting from procedures	Higher
Adoption of anti‐vaccination advice: Increased susceptibility to preventable infections Using vaccine substitutes such as vitamins or homeopathic vaccines	Common with naturopathic and homeopathic beliefs and widely practiced Failure of vaccine substitutes to offer protection from infectious diseases Reduction of herd immunity in the population at large and increased incidence of serious infectious diseases	Indirect: Harm resulting from replacing established standard of care Harm resulting from accepting detrimental health advice beyond the scope of the practitioner's abilities/education/experience	Higher
(c) Ayurvedic medicine			
Toxicity with specific Ayurvedic remedies: Guggul Tablets Sundari Kalp Jambrulin	Numerous reports of lead poisoning, sometimes added or due to manufacturing processes and poor‐quality control. Ayurvedic theory attributes important therapeutic roles to mercury and lead, and many medicines in the Ayurvedic formulary contain at least one metal	Direct: Harm resulting from prescribed substances (including self‐prescribing)	Higher
(d) Religious health advice & faith healing			
Adverse health consequences through acceptance of spiritual health advice that conflicts with medical advice: Conflicting advice from some religious and spiritual groups conflicts with health service provider's advice	Accepting spiritual advice for organic or functional disorders that conflicts with existing effective biomedical treatments may pose a risk to the patient's welfare, for example, in reproductive care, mental health disorders Spiritual advisors treating cognitive‐behavioural or social behaviours as spiritual disorders, for example, autism, homosexuality	Direct or indirect: Harm resulting from reducing the effect of existing medical therapies Harm resulting from accepting detrimental health advice beyond the scope of the practitioner's abilities/education/experience	Higher
3. Physical manipulative alternative health care activities			
(a) Chiropractic			
Injuries resulting from spinal manipulative therapies (SMT): Use of cervical spinal manipulation/adjustment Chiropractic adjustment using forceful SMT procedures such as: Atlas Orthogonal Technique, Activator, Diversified, Korean Specific or Hammer and Chisel Technique	Cerebro‐vascular complications of cervical SMT (vascular dissection, stroke, subdural hematoma) Neurological damage following chiropractic adjustment Fractures or soft‐tissue damage resulting from specific forceful manipulative procedures Preretinal haemorrhages^#^ Tissue damage, for example, myopathy^¤^	Direct: Harm resulting from procedures	Higher Moderate^#^ Lower^¤^
Injuries resulting from SMT in vulnerable specialist populations: Vertebrobasilar accidents or physical injuries in infants and children using chiropractic SMT ‐ particularly of the head and neck High‐velocity thrust SMT in patients with unstable musculo‐skeletal issues Use ofSMT in the elderly^#^ SMT in individuals taking steroids^#^	Spinal/vascular damage resulting in acute embolic events such as transient ischemic attacks (TIAs) and stroke Fractures in patients with skeletal metastasis, rheumatoid arthritis and associated C1/C2 instability and with advanced osteoporosis Spinal/vascular damage Spinal/vascular damage resulting from more friable tissues particularly with long‐term corticosteroid use^#^	Direct: Harm resulting from procedures	Higher Moderate^#^
SMT in individuals with clotting disorders or taking anticoagulants	Increased risk of bleeding and haematoma		Lower
Adoption of anti‐vaccination advice: Increased susceptibility to preventable infections Taking chiropractic advice on chiropractic care as an alternative to vaccination	Common in fundamental chiropractic beliefs and widely practiced Failure of vaccine substitutes to offer protection from infectious diseases Reduction of herd immunity in the population at large and increased incidence of serious infectious diseases	Indirect: Harm resulting from replacing established effective care Harm resulting from accepting advice/treatment beyond the scope of the practitioners educational preparation/training	Higher
(b) Massage therapy			
Injuries with massage therapy in the elderly	Musculo‐skeletal injury due to presence of increased osteoporosis or myopathy	Direct: Harm resulting from procedures	Lower
(c) Osteopathic			
Injuries with prolotherapy Use of prolotherapy injections	Nerve damage due to injections performed near peripheral nerves surrounding joints, tendons and ligaments	Direct: Harm resulting from procedures Harm resulting from accepting detrimental health advice beyond the scope of the practitioner's abilities/education/experience	Moderate
4. Herbal and nutritional alternative therapeutic activities			
Toxicity with specific herbal remedies/supplements that contain metals Remedies containing metals, for example, aluminium, silver, lead, mercury, tin, and zinc	A lack of quality control of metal contaminants is common Metals can build up in body's tissues and cause toxicity, neurological, liver and kidney damage and argyria	Direct: Harm resulting from prescribed substances (including self‐prescribing)	Higher
Toxicity with specific herbal remedies/supplements that are adulterated with other drugs Remedies containing unlisted pharmacological ingredients, for example, arsenic, betamethasone, diazepam	A lack of quality control of pharmacological contaminants is common	Direct: Harm resulting from prescribed substances (including self‐prescribing)	Higher
Adverse effects of specific herbal remedies/supplements: St. John's Wort, Kava, Ginger, Ginkgo, Ginseng, Arnica, Goldenseal, Aloe Vera, Ephedra, Black Cohosh, Feverfew, Henna, Licorice Root, Beta‐carotene†‡	Doses in supplements are much larger than normally orally ingested The labelling of herbal remedies is less regulated, and adverse effects are often unlisted on the packaging	Direct: Harm resulting from prescribed substances (including self‐prescribing)	Higher
Adverse effects of herbal remedies/supplements for weight loss: Containing N‐nitroso‐fenfluramine Containing chromium picolinate	May cause serious hepatotoxicity Several reports of acute nephrotoxicity	Direct: Harm resulting from prescribed substances (including self‐prescribing)	Higher
Adverse effects of herbal remedies/ supplements in vulnerable populations: Use of herbal remedies/supplements recommended for pregnancy‡‡ Use of herbal remedies in the elderly	Oral doses in supplements are much higher than in normal dietary sources May cross placenta and have toxic/teratogenic effects‡‡ ^¤^ May cause premature or complicated labour‡‡ The labelling of herbal remedies is less regulated, and adverse effects in pregnancy may not be listed on the packaging‡‡ ^¤^ Often, research on herbal remedy safety during pregnancy is incomplete‡‡ Toxicity and adverse interactions with reduced metabolism/ excretion in the elderly The labelling of herbal remedies is less regulated, so adverse effects and older adult dosage may not be listed on the packaging	Direct: Harm resulting from prescribed substances (including self‐prescribing)	Higher^¤^ Moderate
Adverse effects of Miracle Mineral Solution (MMS, also known as Master Mineral Solution, or CD protocol) therapies	MMS is a chlorine dioxide solution (a bleach) therapy prescribed by the Genesis II Church of Health and Healing and other alternative practitioners to treat COVID‐19, other infections, acne, cancer, autism and various conditions MMS may cause nausea, vomiting, diarrhoea, tissue damage, acute renal failure and acute hypotension due to dehydration Dosage is not well established in protocols and may be used orally or rectally Often involves distrust of medicine or science or use is influenced by costs or availability of medical treatment	Direct: Harm resulting from prescribed substances (including self‐prescribing)	Higher

*,**,††,§,¤, and # correspond to specific levels of risk identified in column 4.

^†‡^ St. John's Wort: Dermatological sensitivity, headaches, nausea, dizziness, increases the activity of cytochrome P450 enzyme (CYP3A4) and reduces plasma concentrations of certain drugs; Kava: Hepatotoxicity; Ginkgo: Blood clotting; Arnica: Hypertension, Hepatotoxicity; Goldenseal: Bleeding, cardiac arrhythmias, hypotension; Aloe Vera: Cardiac arrhythmias, kidney failure; Ephedra: Hypertension, cardiac arrythmias; Black Cohosh: Hepatotoxicity; Feverfew: Blood clotting; Ginseng: Hypoglycaemia; Ginger: Blood clotting, cardiac arrhythmias. nausea, diarrhoea; Licorice Root: Hypertension; Henna: Dermatological irritation; Beta‐carotene: Increase cancer risk in smokers.

^‡‡^ Alder Buckthorn, Almond Oil, Aloe Vera, Angelica, Anise, Autumn Crocus. Black/Blue Cohosh, Barberry, Beth Root, Bitter Orange, Bloodroot, Bugleweed, Caraway, Cascara, Celery Seeds, Clary Sage, Comfrey, Cotton Bark, Cranberry, Devils Claw, Echinacea, Ephedra, Evening Primrose Oil, Fennel, Fenugreek, Feverfew, Golden Ragwort, Goldenseal, Jasmine, Juniper Berry, Lovage, Mistletoe, Motherwort, Mugwort, Passion Flower, Parsley, Pay D'Arco, Pennyroyal, Peruvian bark, Pulsatilla, Rhubarb, Rosemary, Roman Chamomile, Rue, Saw Palmetto, Saffron, Sage, Sassafras, Shepherds Purse, Thuja, Turmeric, Valerian, Verbena, White Horehound, Wormwood, Yohimbe.

## 4 DISCUSSION

This work revealed specific forms of risk‐associated alternative health practices, but it became clear early in the process that the NCCIH definitions and framework were ineffective as a basis to define or classify the relevant practices (NCCIH, 2018).

### 4.1 Definitions and categorization system

Complementary and alternative medicine is defined by NCCIH as ‘a group of diverse medical and health care systems, practices, and products that are not considered to be part of conventional or allopathic medicine’ and ‘a non‐mainstream practice… used together with conventional medicine’ (NCCIH, 2016, 2018). Although the term is well‐established, there remains considerable debate as to what it actually means in practice, even amongst advocates. In essence, defining what is and is not ‘alternative’ by NCCIH criteria appears more dependent upon the cultural frame of reference than theoretical and practical distinctions, and ‘complementary’ exemplifies this, implying that the intervention is adjunctive to conventional health care practices. Therefore, the term CAM presents a problem in that it (a) conflates alternative with adjunctive strategies and (b) fails to provide a sound rationale for differentiating interventions based upon whether they are used in combination with, or outside of other treatments. Attempting to explain CAM in terms of ‘non‐western scientific and medical traditions’ or ‘allopathic’ proved similarly challenging for the panel, in representing a misleading oversimplification. Another issue with the NCCIH classification was that many of the categorized activities overlapped. For example, some traditional Chinese medicine (TCM) remedies identified are also herbal (and some, herbal versions of biomedical pharmaceutical agents). Also, some classified as mind‐body interventions (e.g., acupuncture) also involved acceptance of an alternative belief framework (NCCIH, 2018). By the fourth Delphi round a consensus had arisen around a functional definition of alternative health care, defined here as:

The range of therapeutics that largely originate from traditions and theories distinct from contemporary biomedical science, and which claim mechanisms of action outside of those currently accepted by scientific and biomedical consensus.

An agreement was also established that the risk‐associated activities identified could simply be categorized under the four specific classes described in Table 3.

### 4.2 Activities identified

#### 4.2.1 General activities

A number of activities were identified that reflected practices unspecific to any particular form of alternative health care. The use of alternative therapies in place of known effective biomedical treatments, for example, may result in illnesses progressing relatively unchecked, or delaying diagnosis, resulting in significant harm including death (Barnes et al., 2008; Johnson et al., 2018b; Lim et al., 2011; Werneke et al., 2004). In a well‐publicized Canadian case, an 11‐year‐old from Ontario died from acute lymphoblastic leukaemia, after she had been sent for treatment to an alternative U.S. clinic in place of chemotherapy (Walker, 2015). Additionally, using newer alternative therapies with unknown or undocumented side effects has been associated with significant adverse events (Anderson et al., 2003; Clarke et al., 2015; Cuthbert et al., 2020; Mackinnon, 2019; Mishori et al., 2011). This issue has also been reported with alternative treatments marketed for COVID‐19 (Freckelton, 2020; Law, 2020).

Another area of concern was use of alternative therapeutics alongside existing medical treatments without informing medical providers, leading to serious health issues due to their combined effects. People often feel uncomfortable telling their doctor they are using alternative health care products, or assume they are harmless, including those used for chronic conditions or during pregnancy (Bahall, 2017; Foley et al., 2019; Nayak et al., 2003; Sprouse & Van Breemen, 2016; Steel et al., 2014; Temple, 2012). One review (Ernst, 2002) found that elderly patients frequently suffered direct harm from alternative therapies and that herbal treatments were associated with serious adverse events through both direct toxicity and drug interactions.

The use of alternative health treatments for non‐medically recognized diagnoses was another area highlighted. Diagnosis may be challenging where signs and symptoms are nonspecific, and in some cases alternative practitioners diagnose conditions that are theoretical, or unjustified. Some common examples are Candida overgrowth/hypersensitivity, adrenal fatigue, chronic Lyme disease and food allergies (Anderson et al., 2001; Cadegiani & Kater, 2016; Gellman, 2020; Lantos, 2015; NIAID, 2010). Psychological harm may arise if clients accept detrimental advice and find themselves in an adversarial position with their medical providers, families and friends. Furthermore, physical harm may arise from employing ineffective therapies or treatments that are damaging, or reduce the effectiveness of other treatments (Cadegiani & Kater, 2016; Jensen et al., 2020; Murdoch et al., 2016). This practice often occurs with the use of alternative health care marketed with deceptive advertising/marketing, another general category of risk identified (Bismark et al., 2018; Fahim et al., 2019; Garrett, Murphy, et al., 2019; Jensen et al., 2020; Murdoch et al., 2016).

Some additional lower risk activities were also identified, including taking part in alternative therapy research, which may not be as well‐regulated as medical or pharmaceutical trials (Turner et al., 2011), and also financial impact to society. The costs of alternative therapies are often significant and may add to health insurance premiums more widely where ineffective alternative health products and services are paid for by third‐party health insurance on the basis of consumer demand (Ostermann et al., 2017; Simpson, 2019; Strahilevitz, 1999).

#### 4.2.2 Alternative belief systems activities

Another risk category reflected activities involving engagement with providers adopting specific alternative health belief systems. These activities were primarily from TCM, naturopathic and Ayurvedic practitioners, although the activities of faith‐healers were also identified. In TCM, a major issue was the toxicity of particular remedies which have resulted in deaths, such as the use of the Aristolochia root for various conditions (Johnson et al., 2018a; Werneke et al., 2004). Despite being banned in many countries now, Aristolochia is still available on the Internet and in some TCM stores (Han et al., 2019; Martena et al., 2007). In one well publicised UK case, a TCM practitioner, prescribed high doses of Aristolochia extracts to treat acne in a 58‐year‐old woman. There is currently no quality scientific evidence supporting Aristolochia use as an effective treatment for acne, or other medical conditions. The woman suffered bilateral renal failure, urinary tract cancer and a myocardial infarction (Holden, 2010).

Quality control in TCM medications has also proved an ongoing concern. The presence of metals and contaminants in several TCM remedies has led to a number of poisoning and deaths (Spilchuk & Thompson, 2019; Tang et al., 2017), and one study found 92% of TCMs examined were found to have some form of contamination and/or substitution (Coghlan et al., 2015). Additionally, injuries had occurred with the practices of cupping and acupuncture, and so were identified of moderate risk (Corado et al., 2019; Huisma, 2015; Jung et al., 2011; Lee et al., 2012; Stenger et al., 2013; Xu et al., 2013). Some lower risk activities with the use of acupuncture in vulnerable populations (the elderly, chronic respiratory disease and immunocompromised) were also noted (Crouch et al., 2001; Ernst, 2002; Lin et al., 2019). Additionally, issues of toxicity of medications were also found with several Ayurvedic preparations (CDC, 2004; Health Canada, 2006; Manohar, 2014; Ontario Agency for Health Protection and Promotion (Public Health Ontario), 2019).

Naturopathic and homeopathic practices were another area where significant risk‐associated practices occurred, particularly the use of invasive therapeutics. In 2017, a 30‐year‐old died as a direct result of an IV Curcumin infection provided by a naturopath in California. The treatment was given for eczema, where a number of safe evidence‐based treatments exist, none involving IV Curcumin. Apart from concerns over the use of inappropriate substances and toxic doses, another noted was that these practitioners had no exposure to acute care or hospital experience in their training and hence were ill‐equipped to deal with acute adverse events that can occur with invasive procedures (FTC, 2018; Hermes, 2017). A number of similar cases have been reported (CDC, 2007; Mackinnon, 2019; US Food & Drug Administration, 2017). Additional areas of concern were the use of colonic therapies (Acosta & Cash, 2009; Mishori et al., 2011) and the high prevalence of anti‐vaccination advice provided by these practitioners (Bleser et al., 2016; Caulfield et al., 2017).

Physical or psychological harm from adopting religiously based health advice conflicting with medical advice was also recognized as a subcategory. Issues of religious directives in reproductive health are probably the best known examples of this (Lentin, 2013; Stephenson et al., 1992). In other well‐documented cases, patients have died as a result of refusing blood transfusions on religious grounds (Hinkson, 2017). Alternative therapies have also been adopted for behaviour, as in the case of Conversion Therapy, where therapeutics are employed under the incorrect assumption that homosexuality is a treatable disorder, resulting in significant psychological problems (Norris, 2018; Ryan et al., 2020). California, New Jersey, Oregon, Illinois, Vermont, Washington, D.C., and, in Canada, Ontario have now passed legislation banning conversion therapy for minors (Drescher et al., 2016).

#### 4.2.3 Physical manipulative therapy activities

The third major class of risk‐associated activities involved manual therapies such as chiropractic, osteopathy and massage. The most serious of these involved chiropractic procedures that resulted in death, serious disability or substantive injury, particularly with cervical vertebral manipulations, or aggressive techniques (such as diversified, activator, Korean or drop‐table procedures). For example, in 2017, a patient attending a chiropractor for leg pain treatment suffered a fractured cervical vertebra, which resulted in his death (Laycock, 2019). Similar cases have been identified over the years, mainly as a result of vascular damage during a procedure, with retinal damage, stroke and sometimes death resulting (Ernst, 2007, 2010; Hufnagel et al., 1999; Jang et al., 2012; Jones et al., 2015; Jumper & Horton, 1996; Lee et al., 1995, 2011; Schmitz et al., 2005; To et al., 2020). Additionally, risks associated with chiropractic treatment in paediatric or elderly populations were also identified, with infant chiropractic noted as a growing trend (Gotlib & Rupert, 2008; Homola, 2010; Humphreys, 2010; Shafrir & Kaufman, 1992; Solheim et al., 2007; Todd et al., 2015). For example, in 2013, an Australian infant suffered a broken neck from a chiropractic manipulation (Medew & Corderoy, 2013). As reporting is very ad hoc, the degree of risk posed by infant and children's chiropractic poses is unknown, but there remains no quality evidence of chiropractic effectiveness for any paediatric condition. Due to the number of reports and severity of injuries, all activities in this category were categorised as higher risk and were noted as one of the most significantly risk‐associated practices. Similar to naturopaths, a high‐prevalence of anti‐vaccination advice has also been provided with chiropractors (Bleser et al., 2016; Davey, 2019; Lombroso, 2015) leading to a directive from the Canadian Chiropractic Association that requests for vaccination advice should be referred to public health authorities and health professionals (CCA, 2019). Lastly, in this group, there were also some reports of less serious adverse events with osteopathic prolotherapy (a more controversial irritant injection technique) deemed of moderate risk (Clifton & Selby, 2018; Dagenais et al., 2010; Krstičević et al., 2017), and even some injuries with massage therapy, also deemed lower risk (Aksoy et al., 2009; Hsu et al., 2017; Humphreys, 2010).

#### 4.2.4 Herbal and nutritional supplement therapies

The final class identified was that of alternative herbal and nutritional therapies, primarily with toxicity and quality‐control issues. Similar to TCM remedies, contamination with metals has been well‐documented (Buettner et al., 2009; Ernst, 1998; Locatelli et al., 2014; Sakharkar, 2017; Saper et al., 2004). Additionally, many supplements contain unlisted ingredients, including other drugs (Sovak et al., 2002; Steinhoff, 2019; Zhang et al., 2012). One study found 33% of products tested contained unlisted ingredients (Newmaster et al., 2013), whilst a 2019 study reported that 27% of the herbal products in the global marketplace were adulterated (Ichim, 2019). Many commercially available herbal remedies also have toxic effects when used in larger doses, or in certain circumstances (Chen et al., 2013; Cohen & Ernst, 2010; De Groot, 2013; Ernst, 2000; Fu et al., 2008; Lee et al., 2020; Lynch et al., 2006; McEwen, 2015; Mei et al., 2017; Nazari et al., 2017; Odaguchi et al., 2019; The Alpha‐Tocopherol Beta Carotene Cancer Prevention Study Group, 1994; Venkatramani et al., 2013; Werneke et al., 2004). Herbal products are usually controlled under different regulations to drugs (e.g., as dietary supplements or natural health products) and have not been subjected to the same scientific scrutiny. Hence, they are not required to meet the higher standards required for pharmaceutical products and packaging frequently lacks information on safe dosage, toxicity or contra‐indications (Ekor, 2014).

Supplements are frequently marketed for weight loss, and toxicity has been demonstrated when people consume larger doses. Additionally, many herbal products used during pregnancy and in the elderly have well‐documented problems with serious adverse outcomes (Adachi et al., 2003; Cerulli et al., 1998; Gabardi et al., 2007; Kanda et al., 2003; Patel et al., 2012; Siska, 2017; Vincent, 2003). Lastly in this category, the use of oral sodium chlorite/chlorine dioxide solutions as supplements to treat autism and other conditions (including COVID‐19) was recognized as having become popular recently, with associated serious adverse inflammatory events (Anon, 2020; FDA, 2020; Health Canada, 2018; Loh & Shafi, 2014). Overall, this suggests that common assumptions that herbal and supplement products are safe and well‐regulated are erroneous, and while adverse events may be rare, effects can be very harmful, and the risk of consumption weighed against questionable evidence of efficacy.

### 4.3 Relative risk and informed consent

Aside from the health professions, the major stakeholders in health care are patients/clients, families, caregivers, communities, government/regulatory health agencies, third‐party payers such as insurance companies, pharmaceutical and health product businesses, unions and employers. All of these have an interest in establishing the safety of alternative health care interventions, and the implementation of improved and mandatory reporting systems for associated adverse events would help quantitatively establish the risks involved so that relative risk may be established. Nevertheless, as the efficacy of alternative health care interventions remain empirically undemonstrated, those that pose a significant risk of harm should be regarded as *higher risk* activities for the general public, and even those categorized as *moderate risk* are best treated with caution. Any comparison of these adverse events to biomedical practice is also likely unhelpful, as this involves a false equivalency, with both the levels of acuity of illness and adverse event monitoring involved.

Lastly, it is also worth considering the role of informed consent and the responsibilities of those providing alternative health care interventions to make sure that the client is fully informed of the potential risks involved, even if the incidence is unknown. Few of the practices described here are provided with open information regarding the potential hazards, and as some risk severe harm.

### 4.4 Limitations

As the nature of alternative health care is dynamic, this work simply represents a review of current activity. A limitation is that we acknowledge the efficacy of the therapies explored remains scientifically undemonstrated and incidence of adverse events unknown as the data is unavailable. Given that, risks are contrasted against seriousness of adverse events rather than incidence. Additionally, this qualitative exploratory approach reflects the findings of a group of health care professionals in specific domains, which may not be more widely generalisable. This is not a comparative study but represents an initial attempt to develop a simple taxonomic framework that requires further testing and refinement.

## 5 CONCLUSIONS

This study confirmed that established definitions of CAM/health care are problematic and impractical for classifying them and exploring potential associated risks; a more practical definition is provided to address this. A significant number of risk‐associated practices related to alternative health care were identified. Broadly, these were categorized into general practices that conflict with biomedical care or involved largely untested therapies, those involving alternative beliefs systems, physical manipulative alternative therapies and herbal and nutritional supplements. Some risk significant harms including major physical injuries or even death. The lack of systematic methods for recording adverse events in alternative health care also makes establishing the frequency of such events challenging. However, it is important that people engaging with alternative health care understand they are not necessarily risk‐free endeavours and what those risks are.

## Data Availability

The data that support the findings of this study are available on reasonable request from the corresponding author. The data are not publicly available due to privacy or ethical restrictions.
